# Supplementary material for: A human monoclonal antibody binds within the poliovirus receptor-binding site to neutralize all three serotypes
Source: Nat Commun. 2023 Oct 10;14:6335. doi: 10.1038/s41467-023-41052-9 (PMC10564760; doi:10.1038/s41467-023-41052-9)
Supplement: Supplementary file 1 — Supplementary information [file 41467_2023_41052_MOESM1_ESM.pdf]

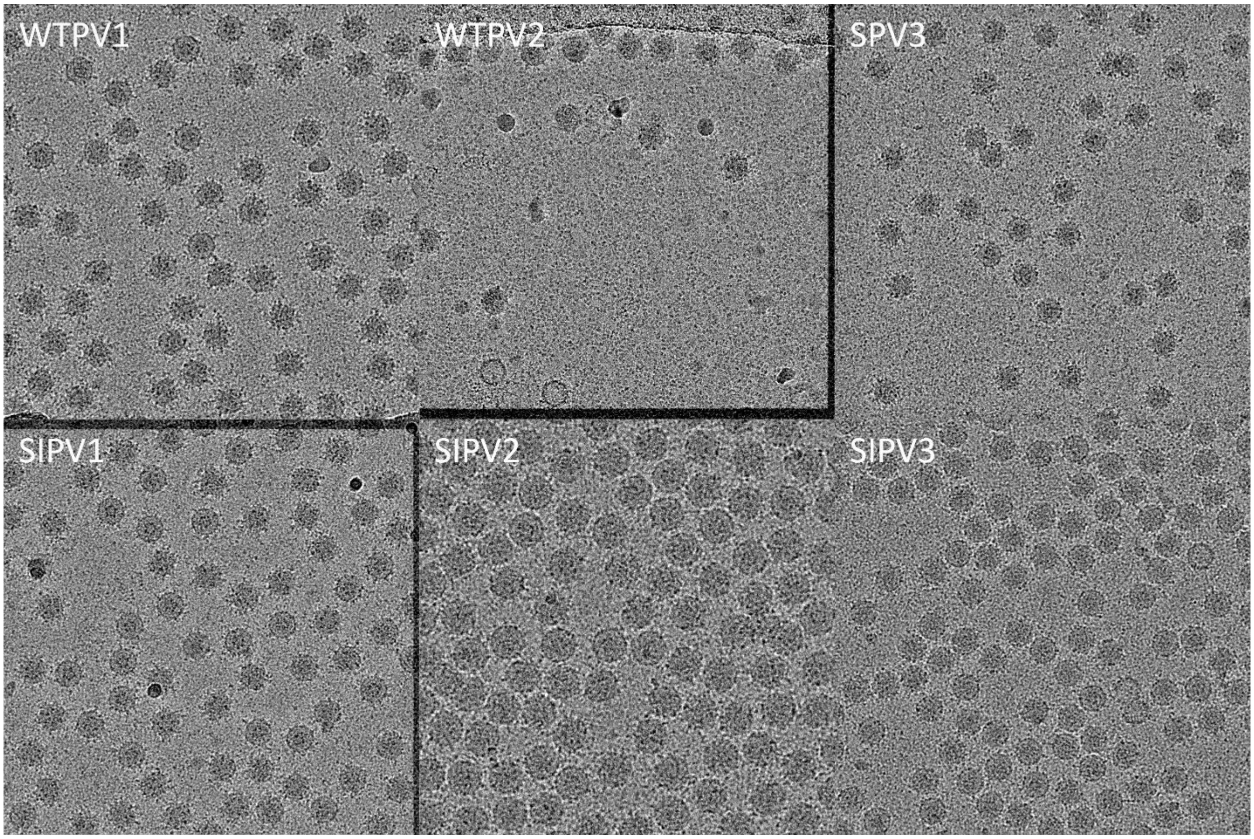

Supplemental Figure 1: Motion Corrected Micrographs. Example representative motion corrected micrographs are shown. Fab is visible decorating the viral capsids. Central densities represents genome.

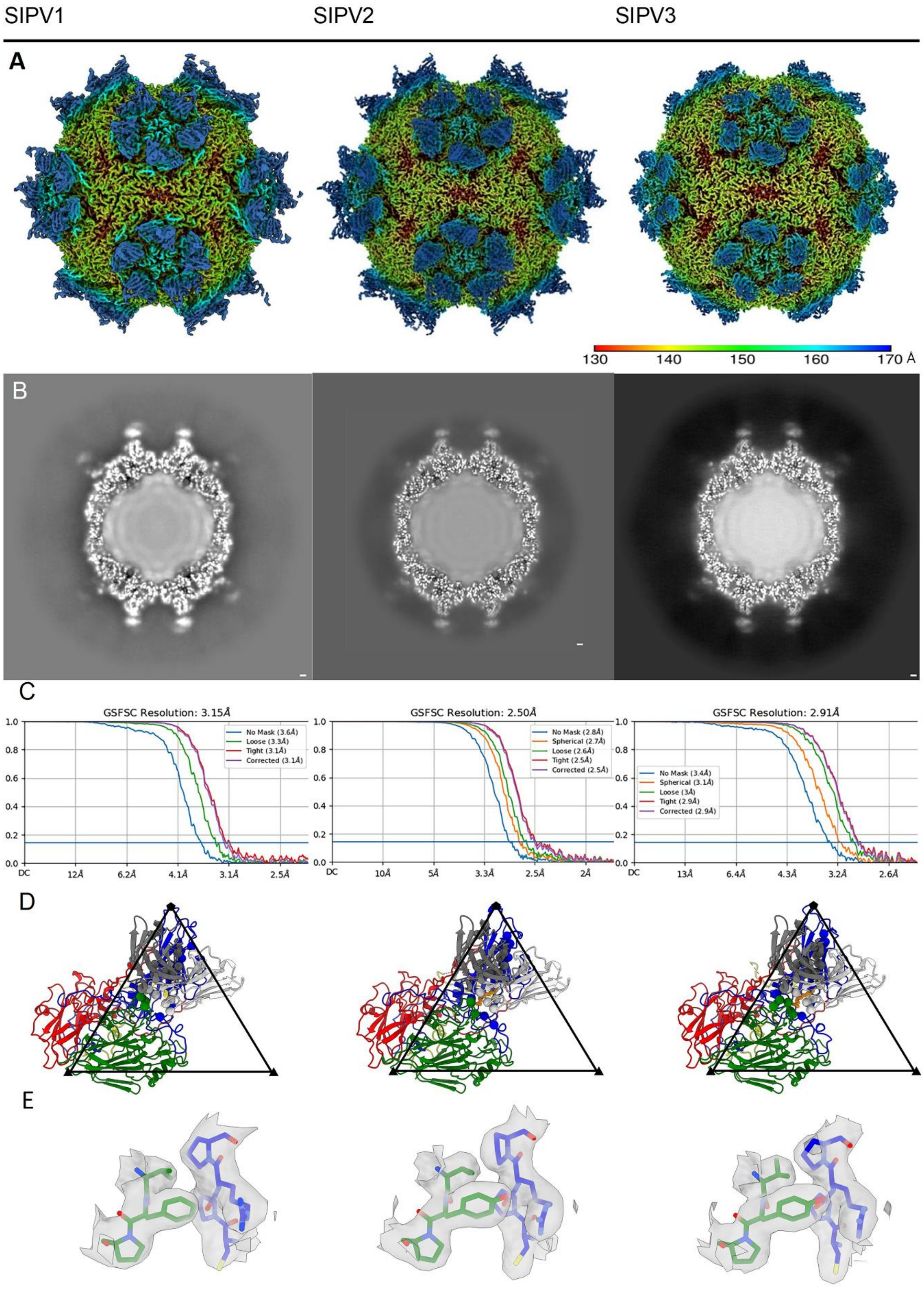
SIPV1
SIPV2
SIPV3
A
130
140
150
160
170 Å
B
C
GSFSC Resolution: 3.15Å
No Mask (3.6Å)
Loose (3.3Å)
Tight (3.1Å)
Corrected (3.1Å)
GSFSC Resolution: 2.50Å
No Mask (2.8Å)
Spherical (2.7Å)
Loose (2.6Å)
Tight (2.5Å)
Corrected (2.5Å)
GSFSC Resolution: 2.91Å
No Mask (3.4Å)
Spherical (3.1Å)
Loose (3Å)
Tight (2.9Å)
Corrected (2.9Å)
D
E

Supplemental Figure 2: Inactivated Fab-virus maps: 9H2 Fab binds virus with same mode of binding in all three serotypes (inactivated virus): A: Surface rendered DeepEMhancer sharpened maps are colored according to radius (color key) and show density corresponding to the 9H2 Fab. **B:** The central section shows the Fab has similar magnitude of density as the capsid. **C:** FSC curves indicate resolution ranging from 2.5 – 3.2 Å at the gold standard 0.143 cutoff. **D:** Refined models of single virus protomer (VP1-4; blue, green, red, yellow) and bound 9H2 variable domain (heavy and light chain; dark and light gray). SIPV1, 2 and 3 data were taken at magnification 59000x, 75000x, and 59000x resulting in pixel sizes of 1.1, 0.89, and 1.1 respectively. **E:** For each complex, a representative area illustrating the quality of the model built into the map is shown. The map area highlighted includes residues VP1 270-273 and VP2 192-194. Coloration is VP1 (blue) and VP2 (green) with additional coloring by heteroatom.

SIPV1

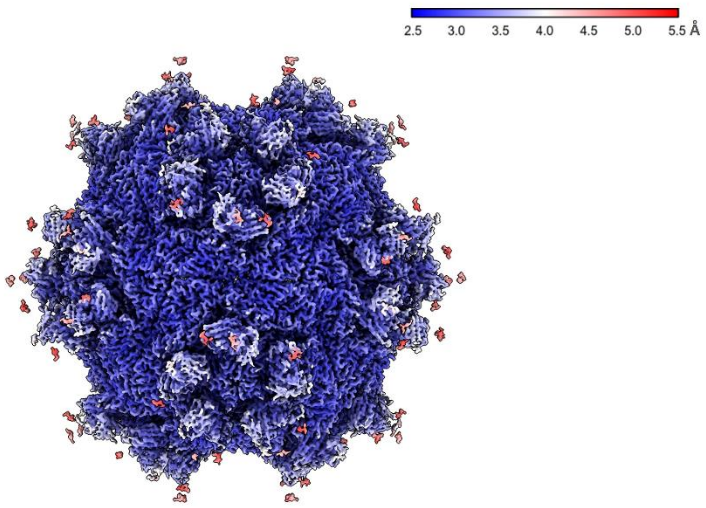

SIPV2

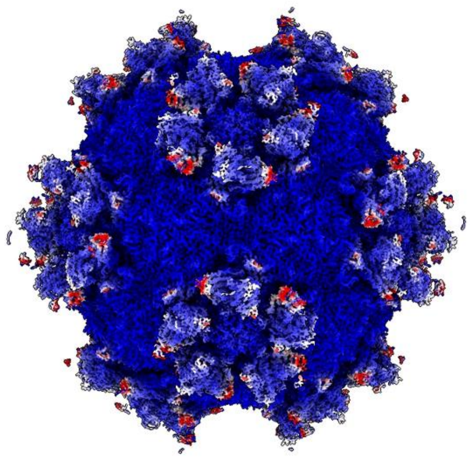

SIPV3

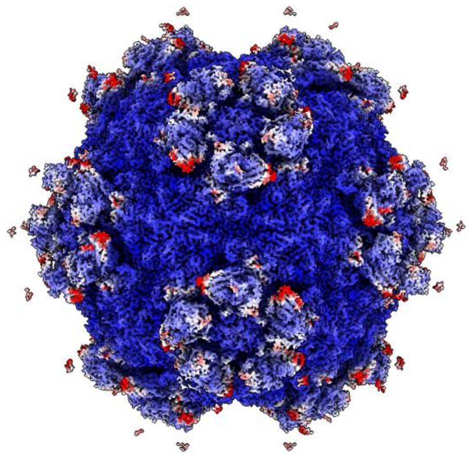

Supplemental Figure 3: Local Resolution Maps: Local resolution values were measured in Angstroms and displayed with surface rendered for each inactive virus-Fab complex. The best resolution is present in the capsid shell while the poorest is found in the hinge region of the Fab fragments.

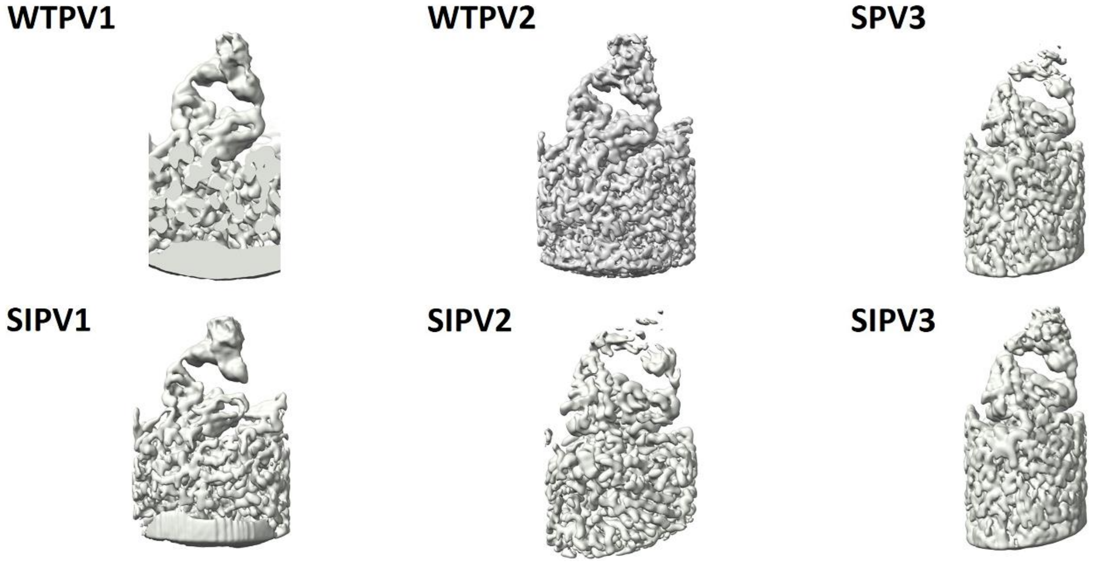

Supplemental Figure 4: 9H2 Subparticles: Examples of subparticle classes for each virus complex with 9H2 are shown. Multiple subparticle classes were merged for the final maps which were recombined to produce a symbreak full icosahedral map into which virus and 9H2 were built (Methods).

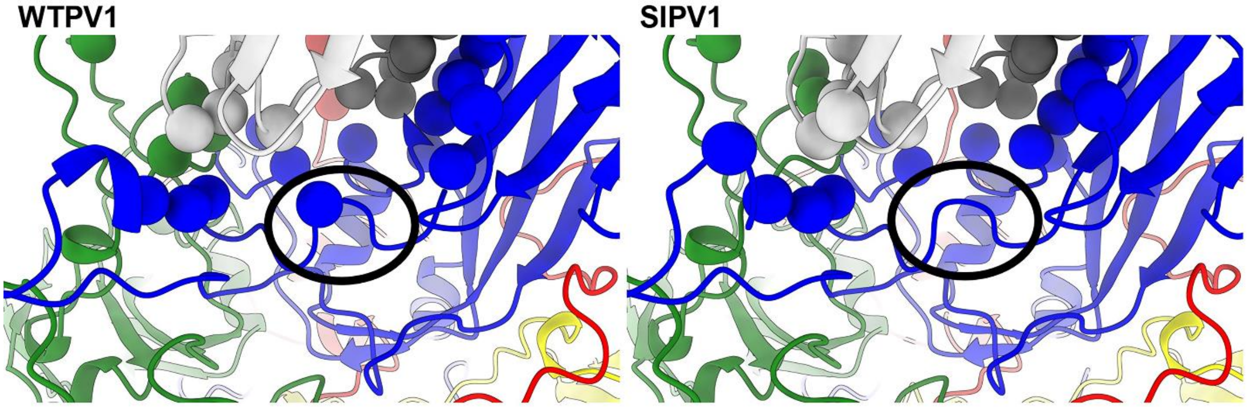

Supplemental Figure 5: Canyon Floor Loop: Unlike the crystal structure 1HXS, between wild type and inactivated Sabin, the structure and conformation of the loop (black circle) comprised of VP1 residues 232-238 superimpose. Residues identified as contacts are displayed as spheres matching their chain color (heavy and light chain; dark and light gray; VP1-3; blue, green, red).

```
VP1 contacts 18-21
ISPV1  60   TVQTRHVVQHRSRSESSIESFFARGACVAIITVDNSASTKNKDKLFTVWKITYKDTVQLR    119
WTPV1  60   TVQTRHVVQHRSRSESSIESFFARGACVTIMTVDNPASTTNKDKLFAVWKITYKDTVQLR    119
ISPV2  60   TVQTRHVIQRRTRSESTVESFFARGACVAIIEVDNDAPTKRASRLFSVWKITYKDTVQLR    119
WTPV2  60   TVQTRHVIQKRTRSESTVESFFARGACVAIIEVDNDAPTKRASKLFSVWKITYKDTVQLR    119
ISPV3  60   TVQTRHVVQRRSRSESTIESFFARGACVAIIEVDNEQPTTRAQKLFAMWRITYKDTVQLR    119
SPV3   60   TVQTRHVVQRRSRSESTIESFFARGACVAIIEVDNEQPTTRAQKLFAMWRITYKDTVQLR    119
            *******:*:*:****::**********:*: ***   *.. .:**::*:**********

ISPV1 120   RKLEFFTYSRFDMEFTFVVTANFTETNNGHALNQVYQIMYVPPGAPVPEKWDDYTWQTSS    179
WTPV1 120   RKLEFFTYSRFDMELTFVVTANFTETNNGHALNQVYQIMYVPPGAPVPEKWDDYTWQTSS    179
ISPV2 120   RKLEFFTYSRFDMEFTFVVTSNYIDANNGHALNQVYQIMYIPPGAPIPGKWNDYTWQTSS    179
WTPV2 120   RKLEFFTYSRFDMEFTFVVTSNYTDANNGHALNQVYQIMYIPPGAPIPGKWNDYTWQTSS    179
ISPV3 120   RKLEFFTYSRFDMEFTFVVTANFTNANNGHALNQVYQIMYIPPGAPTPKSWDDYTWQTSS    179
SPV3  120   RKLEFFTYSRFDMEFTFVVTANFTNANNGHALNQVYQIMYIPPGAPTPKSWDDYTWQTSS    179
            **************:*****:*: ::**************:***** * .*:********

ISPV1 180   NPSIFYTYGTAPARISVPYVGISNAYSHFYDGFSKVPLKDQS-AALGDSLYGAASLNDFG    238
WTPV1 180   NPSIFYTYGTAPARISVPYVGISNAYSHFYDGFSKVPLKDQS-AALGDSLYGAASLNDFG    238
ISPV2 180   NPSVFYTYGAPPARISVPYVGIANAYSHFYDGFAKVPLAGQA-STEGDSLYGAASLNDFG    238
WTPV2 180   NPSVFYTYGAPPARISVPYVGIANAYSHFYDGFAKVPLAGQA-STEGDSLYGAASLNDFG    238
SIPV3 180   NPSIFYTYGAAPARISVPYVGLANAYSHFYDGFAKVPLKTDANDQIGDSLYSAMTVDDFG    239
SPV3  180   NPSIFYTYGAAPARISVPYVGLANAYSHFYDGFAKVPLKTDANDQIGDSLYSAMTVDDFG    239
            ***:*****: **********::**********:****   ::     *****.* :::***

ISPV1 239   ILAVRVVNDHNPTKVTSKIRVYLKPKHIRVWCPRPPRAVAYYGPGVDYKDGTLTPLSTKD    298
WTPV1 239   ILAVRVVNDHNPTKVTSKIRVYLKPKHIRVWCPRPPRAVAYYGPGVDYKDGTLTPLSTKD    298
ISPV2 239   SLAVRVVNDHNPTRLTSKIRVYMKPKHVRVWCPRPPRAVPYFGPGVDYKDG-LTPLPEKG    297
WTPV2 239   SLAVRVVNDHNPTKLTSKIRVYMKPKHVRVWCPRPPRAVPYYGPGVDYKDG-LAPLPGKG    297
ISPV3 240   VLAVRVVNDHNPTKVTSKVRIYMKPKHVRVWCPRPPRAVPYYGPGVDYRNN-LDPLSEKG    298
SPV3  240   VLAVRVVNDHNPTKVTSKVRIYMKPKHVRVWCPRPPRAVPYYGPGVDYRNN-LDPLSEKG    298
             ************::***:*:*:****:*********** *:******::. * **  *.


VP2 contacts 3-6
ISPV1 121   ALGVFAVPEMCLAGDSNTTTMHTSYQNANPGEKGGTFTGTFTPDDNQTSPARRFCPVDYL    180
WTPV1 121   ALGVFAVPEMCLAGDSNTTTMHTSYQNANPGEKGGTFTGTFTPDNNQTSPARRFCPVDYL    180
ISPV2 121   ALGVFAVPEMCLAGDST-THMFTKYENANPGEKGGEFKGSFTLDTNATNPARNFCPVDYL    179
WTPV2 121   ALGVFAVPEMCLAGDST-THMFTKYENANPGEKGGEFKGSFTLDTNATNPARNFCPVDYL    179
ISPV3 121   ALGVFAIPEYCLAGDSDK-QRYTSYANANPGERGGKFYSQFNKDNAVTSPKREFCPVDYL    179
SPV3  121   ALGVFAIPEYCLAGDSDK-QRYTSYANANPGERGGKFYSQFNKDNAVTSPKREFCPVDYL    179
            ******:** ******      .*.* ******:** * . *. *   *.* *.*******


VP3 contacts 1-2
ISPV1 226   RDTTHIEQKA    235 3.15 Å
WTPV1 226   RDTTHIEQKA    235 3.13
ISPV2 226   RDTTHISQEA    235 2.50
WTPV2 226   RDTTHISQEA    235 2.73
ISPV3 226   RDTTHISQSA    235 2.91
SPV3  226   RDTTHISQSA    235 2.66


TOTAL 9H2 CONTACTS: 23-27
```

Supplemental Figure 6: Sequence Alignment of Polioviruses: 9H2 contacts are highlighted in this Clustal Omega sequence alignment. Contact number is provided as low – high end estimates for 9H2 footprint residue contacts. Variation likely resulted from differences in resolution among maps and slight differences in chain and rotamer placement.

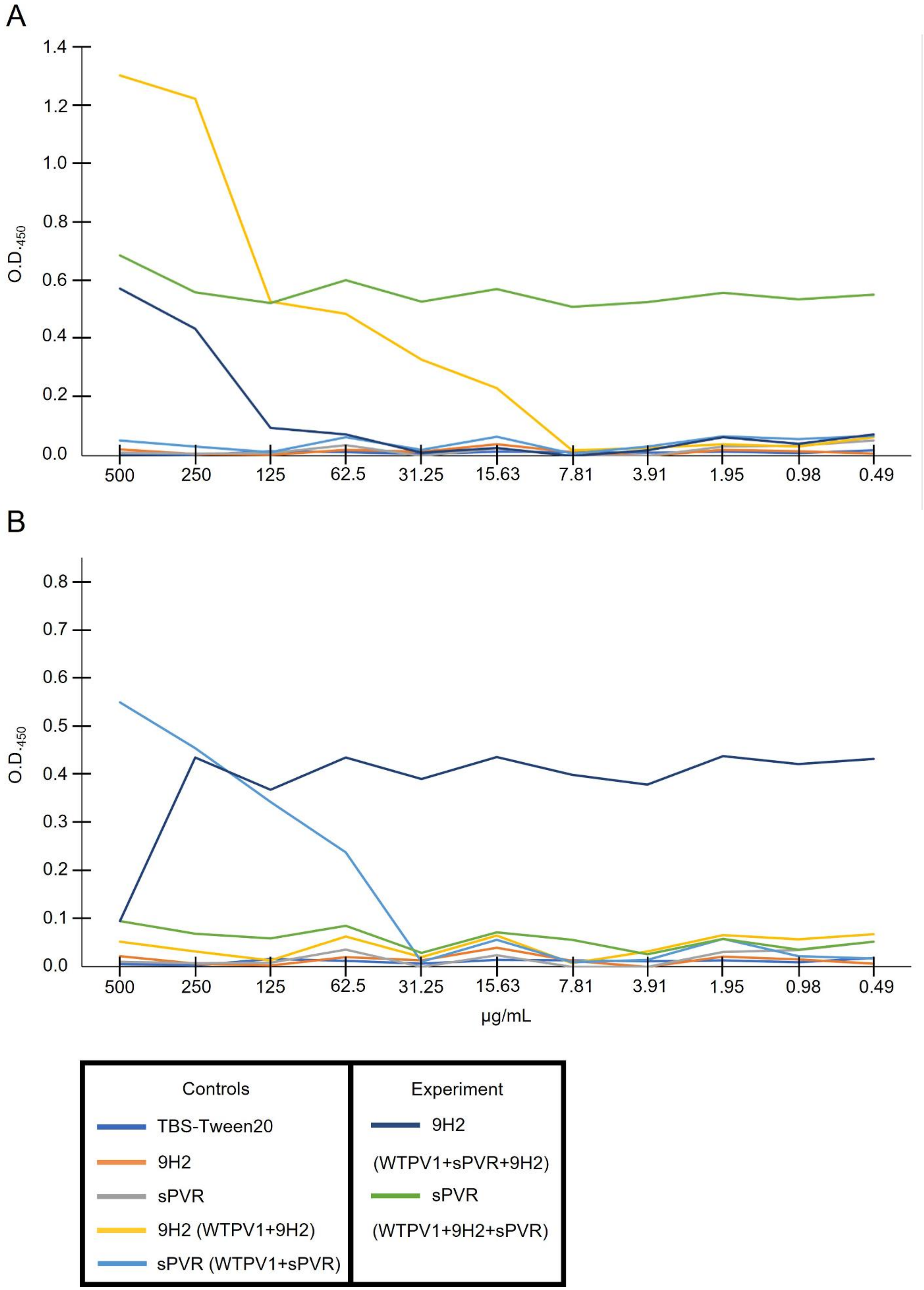
A
1.4
1.2
1.0
0.8
0.6
0.4
0.2
0.0
O.D.450
500
250
125
62.5
31.25
15.63
7.81
3.91
1.95
0.98
0.49
B
0.8
0.7
0.6
0.5
0.4
0.3
0.2
0.1
0.0
O.D.450
µg/mL
Controls
TBS-Tween20
9H2
sPVR
9H2 (WTPV1+9H2)
sPVR (WTPV1+sPVR)
Experiment
9H2
(WTPV1+sPVR+9H2)
sPVR
(WTPV1+9H2+sPVR)

Supplemental Figure 7: Binding Competition ELISA: Binding of human monoclonal antibody 9H2 or recombinant soluble poliovirus receptor (sPVR) to wild type poliovirus type 1(Mahoney)(WTPV1) or WTPV1 complexed with 9H2 or sPVR. Binding was assessed by enzyme-linked immunosorbent assay (ELISA) and reported optical density at 450nM. The results are representative of three independent experiments. X axes are the concentration of 9H2 mAb or sPVR in µg/mL. Y axes are optical density readings at 450 nm. Color key labels indicate the specimen being serially diluted and controls. For both A and B the green line indicates the ability of sPVR to compete off bound 9H2 by plotting the results of the sequential addition of virus, 9H2 held constant at 0.1 mg/mL across all dilution wells, followed by PVR at various dilutions. For both A and B the blue line represents the ability for 9H2 to outcompete receptor; whereby the treatment corresponds to the sequential addition of PV1, followed by the addition of a consistent amount (0.1 mg/mL) of sPVR, and final addition of 9H2 at various dilutions. **A:** Human mAb-HRP secondary antibody was used to report 9H2 bound to captured virus. **B:** Anti-sPVR secondary mAb-HRP was used to report sPVR bound to captured virus. Application of 9H2 mAb at 0.1 mg/mL is capable of preventing subsequent sPVR (at 0.5 mg/mL) binding to the virus whereas 0.5 mg/mL 9H2 mAb can outcompete previously bound sPVR (at 0.1 mg/mL).

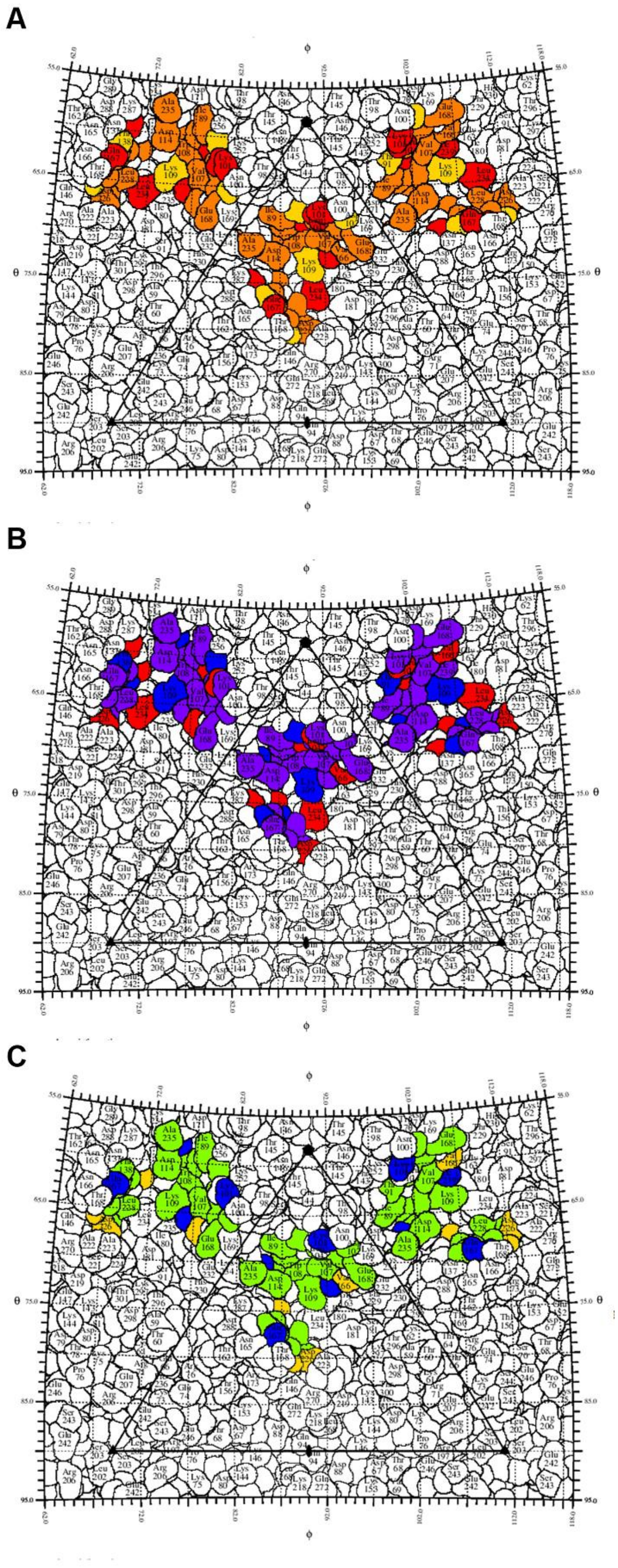

Supplemental Figure 8: Live Footprint Comparisons: A-C: 9H2 footprint comparisons among live PVs showing differences between serotypes. PV1 (Red), PV2 (gold), PV3 (blue). Overlaps shown; PV1 & PV2, orange; PV1 & PV3, purple; PV2 & PV3, green.

Supplemental Table 1: Data Collection, Processing, and Refinement Statistics

**Data Collection and Processing**	WTPV1	WTPV2	SPV3	SIPV1	SIPV2	SIPV3
magnification	59k	59k	120k	59k	75k	59k
voltage (kV)	300	300	200	300	300	300
e^-/Å^2	40	40	50	40	40	40
defocus range (μm)	1-3	1-3	0.5-2	1-2.5	0.75-2.5	0.75-2.5
pixel size (Å)	1.1	1.1	1.2	1.1	0.86	1.1
symmetry imposed	ICOS	ICOS	ICOS	ICOS	ICOS	ICOS
initial micrographs	1592	2284	3737	2302	1921	734
final micrographs	1550	2185	2915	1883	1701	714
particles	79471	11599	84951	99462	111182	52115
map resolution (Å)	3.1	2.7	2.7	3.2	2.5	2.9

**Refinement**						
model resolution* d_{model} (Å)	3.3	2.9	2.8	3.3	2.6	3.0
model resolution** $d_{FSC\ model}$ 0.143 (Å)	3.1	2.7	2.6	3.1	2.5	2.8
map sharpening B factor	176.3	109.3	135.5	181.3	129.5	145.5
non-hydrogen atoms	8318	8245	8343	8300	8310	8329
protein residues	1068	1059	1068	1064	1066	1066
ligands	N/A	PLM	PLM	N/A	PLM	PLM
protein B factors mean (Å2)	100.83	58.74	57.55	91.93	54.20	73.54
ligand B factors (Å2)	N/A	58.98	66.86	N/A	55.04	70.11
RMS deviation bond length (Å)	0.005	0.006	0.007	0.004	0.005	0.005
RMS deviation bond angle (˚)	0.774	0.898	1.083	0.712	0.792	0.893
MolProbity score	0.95	1.20	1.11	1.00	1.13	1.31
Clashscore	0.67	1.78	0.67	1.47	1.77	1.52
Rotamer outliers (%)	0.11	1.55	1.74	1.41	0.44	1.74
Ramachandran favored (%)	96.49	97.41	96.67	98.00	96.77	96.48
Ramachandran allowed (%)	3.42	2.49	3.33	2.00	3.23	3.52
Ramachandran outliers (%)	0.09	0.10	0.00	0.00	0.00	0.00

Common statistics and parameters from the six 9H2 Fab-poliovirus complexes, with refinement statistics generated from Phenix comprehensive validation. ICOS=icosahedral. PLM=palmitic acid.

*Model resolution d_{model} is the resolution of the map calculated from the final model that maximizes the correlation to the experimental map[1].
**Model resolution d_{FSC_model} is the resolution cutoff at which the model and experimental map Fourier coefficients are most similar[1].

Supplemental Table 2: Capsid Contacts

WTPV1

VP1	87 88 89 90 101 102 104 105 106 107 108 114 166 168 226 227 228 234 239 280 282
VP2	139 140 142 167
VP3	233 235

SIPV1

VP1	87 88 89 90 101 104 105 106 107 108 109 114 166 168 169 223 226 227 228 280 282
VP2	138 139 140 167
VP3	235

WTPV2

VP1	87 88 89 91 102 103 105 106 107 108 109 114 166 168 226 227 228 282
VP2	137 138 139 141 171
VP3	233 235

SIPV2

VP1	87 88 89 90 100 101 103 105 106 107 108 114 166 168 228 234 239 282
VP2	137 138 139 141
VP3	235

SPV3

VP1	87 88 89 91 101 102 103 105 106 107 108 109 114 115 168 228 229 240
VP2	137 138 139 140 141 166
VP3	233 235

SIPV3

VP1	87 88 89 91 100 101 102 103 105 106 107 108 114 168 224 228 229 240 281
VP2	137 140 141
VP3	235

Identified contact residue numbers in each 9H2-poliovirus complex for each virus chain.

Supplemental Table 3: 9H2 Contacts

WTPV1	
Heavy chain	81 119 120 121 122 123 124 125 127 128 129 130
Light chain	45 46 50 73 74 114 115

WTPV2	
Heavy chain	74 121 122 123 124 125 127 128 129 130 131
Light chain	45 46 50 74 114 115

SPV3	
Heavy chain	74 81 120 121 122 123 124 125 127 128 129 130 131
Light chain	44 45 46 50 52 73 74 114 115

SIPV1	
Heavy chain	81 119 120 121 122 123 124 125 127 128 129 130
Light chain	45 46 50 73 74 75 90 113 114 115

SIPV2	
Heavy chain	46 120 121 123 124 125 127 128 129 130 131
Light chain	45 46 49 50 74 114 115

SIPV3	
Heavy chain	46 120 121 122 123 124 125 127 128 129 130 131
Light chain	45 46 50 73 74 90 115

Identified contact residue numbers in each 9H2-poliovirus complex for each Fab chain.

Supplemental Table 4: 9H2 Fab Binding Constants

	KD (M)	Ka (1/Ms)	Ka error	Kd (1/s)	Kd error
SPV1	9.364×10^{-4}	1.122×10^{3}	8.470×10^{4}	1.050	0.1795
SIPV2	2.097×10^{-6}	2.301×10^{5}	1.182×10^{5}	0.4826	0.1374
SPV3	3.232×10^{-6}	1.461×10^{5}	6.838×10^{4}	4.769×10^{-2}	4.769×10^{-2}

9H2 Fab binding constants as provided by analysis of biolayer interferometry data.

Supplemental References:

1) Afonine, P. V., Klaholz, B. P., Moriarty, N. W., Poon, B. K., Sobolev, O. V., Terwilliger, T. C., Adams, P. D., & Urzhumtsev, A. (2018). New tools for the analysis and validation of cryo-EM maps and atomic models. *Acta Crystallographica Section D Structural Biology*, *74*(9), 814–840. https://doi.org/10.1107/S2059798318009324
