# Supplementary material for: A human monoclonal antibody binds within the poliovirus receptor-binding site to neutralize all three serotypes
Source: Nat Commun. 2023 Oct 10;14:6335. doi: 10.1038/s41467-023-41052-9 (PMC10564760; doi:10.1038/s41467-023-41052-9)
Supplement: Supplementary file 3 — Description of Additional Supplementary files [file 41467_2023_41052_MOESM3_ESM.pdf]

File name: Supplementary Movie 1

Description: Fab binds into and across the canyon. WTPV1 and 9H2 Fab final models are shown as surface and ribbon respectively. The end of the movie highlights just how far into the canyon the heavy chain CDR 3 reaches. Chain colors: heavy and light chain; dark and light gray; VP1-3; blue, green, red.
